# Hypoxia Increases the Potential for Neutrophil-mediated Endothelial Damage in Chronic Obstructive Pulmonary Disease

**DOI:** 10.1164/rccm.202006-2467OC

**Published:** 2022-01-19

**Authors:** Katharine M. Lodge, Arlette Vassallo, Bin Liu, Merete Long, Zhen Tong, Paul R. Newby, Danya Agha-Jaffar, Koralia Paschalaki, Clara E. Green, Kylie B. R. Belchamber, Victoria C. Ridger, Robert A. Stockley, Elizabeth Sapey, Charlotte Summers, Andrew S. Cowburn, Edwin R. Chilvers, Wei Li, Alison M. Condliffe

**Affiliations:** ^1^ Department of Medicine, School of Clinical Medicine, University of Cambridge, Cambridge, United Kingdom;; ^2^ National Heart and Lung Institute, Imperial College London, London, United Kingdom;; ^3^ Department of Infection, Immunity, and Cardiovascular Disease, University of Sheffield, Sheffield, United Kingdom; and; ^4^ Institute of Inflammation and Ageing, University of Birmingham and; ^5^ University Hospitals Birmingham NHS Foundation Trust, Queen Elizabeth Hospital Birmingham, Birmingham, United Kingdom

**Keywords:** cell degranulation, neutrophil elastase, vascular endothelium, cardiovascular disease

## Abstract

**Rationale:**

Patients with chronic obstructive pulmonary disease (COPD) experience excess cardiovascular morbidity and mortality, and exacerbations further increase the risk of such events. COPD is associated with persistent blood and airway neutrophilia and systemic and tissue hypoxia. Hypoxia augments neutrophil elastase release, enhancing capacity for tissue injury.

**Objective:**

To determine whether hypoxia-driven neutrophil protein secretion contributes to endothelial damage in COPD.

**Methods:**

The healthy human neutrophil secretome generated under normoxic or hypoxic conditions was characterized by quantitative mass spectrometry, and the capacity for neutrophil-mediated endothelial damage was assessed. Histotoxic protein concentrations were measured in normoxic versus hypoxic neutrophil supernatants and plasma from patients experiencing COPD exacerbation and healthy control subjects.

**Measurements and Main Results:**

Hypoxia promoted PI3Kγ-dependent neutrophil elastase secretion, with greater release seen in neutrophils from patients with COPD. Supernatants from neutrophils incubated under hypoxia caused pulmonary endothelial cell damage, and identical supernatants from COPD neutrophils increased neutrophil adherence to endothelial cells. Proteomics revealed differential neutrophil protein secretion under hypoxia and normoxia, and hypoxia augmented secretion of a subset of histotoxic granule and cytosolic proteins, with significantly greater release seen in COPD neutrophils. The plasma of patients with COPD had higher content of hypoxia-upregulated neutrophil-derived proteins and protease activity, and vascular injury markers.

**Conclusions:**

Hypoxia drives a destructive “hypersecretory” neutrophil phenotype conferring enhanced capacity for endothelial injury, with a corresponding signature of neutrophil degranulation and vascular injury identified in plasma of patients with COPD. Thus, hypoxic enhancement of neutrophil degranulation may contribute to increased cardiovascular risk in COPD. These insights may identify new therapeutic opportunities for endothelial damage in COPD.

**At a Glance Commentary**

**Scientific Knowledge on the Subject**

Chronic obstructive pulmonary disease (COPD) is characterized by persistent neutrophilia in the setting of local and systemic hypoxia and is associated with excess cardiovascular disease, even allowing for known risk factors. Neutrophils accumulating in areas of inflammation and microcirculatory impairment experience profound hypoxia, which prolongs their survival and increases their secretory responses. Thus, hypoxic neutrophils have increased potential to cause endothelial injury, but their role in mediating the increased cardiovascular risk in COPD is poorly understood.

**What This Study Adds to the Field**

Herein we show that hypoxia augments the ability of neutrophils to selectively secrete a subset of histotoxic proteins capable of damaging endothelial cells. Hypoxia further enhanced release of these proteins from neutrophils from patients experiencing COPD exacerbation, with elevated concentrations also detected in patient plasma. This study suggests that hypoxic enhancement of neutrophil degranulation may contribute to increased cardiovascular risk in COPD and that the hypoxic neutrophil secretome proteins may represent new therapeutic targets to alleviate endothelial dysfunction in COPD.

Chronic obstructive pulmonary disease (COPD) is characterized by neutrophilic inflammation in the setting of tissue (and often systemic) hypoxia and by increased risk of cardiovascular disease and pulmonary hypertension. Neutrophil elastase (NE) has been implicated in COPD pathogenesis (1), and we have previously shown that hypoxia markedly augments NE release from neutrophils to promote respiratory epithelial cell damage (2). However, the impact of hypoxia on the extended neutrophil secretome and the potential for “hypoxic neutrophils” to injure other disease-relevant cell types are currently unknown. Identifying novel targets implicated in driving COPD morbidity may provide new therapeutic opportunities.

Even in health, certain tissues (e.g., muscle) are hypoxic (3). This “physiological hypoxia” may be compounded during exercise, inducing neutrophil phenotypic changes (4). In disease, profound “pathological hypoxia” exists in inflamed or infected tissues and areas of microcirculatory impairment. Although patients with severe COPD are systemically hypoxic, significant tissue hypoxia (<1.3% oxygen) can occur even in mild disease, demonstrated in inflamed airways (5–7) and atherosclerotic vasculature (8), where neutrophils accumulate. Upregulation of hypoxia-inducible factors in neutrophils from patients with acute lung injury, including coronavirus disease (COVID-19) infection, indicates neutrophil exposure to hypoxia *in vivo* (9).

Neutrophil antimicrobial function depends on the fusion of preformed granules, containing cytotoxic proteins and proteases, with the pathogen-containing phagosome. However, highly activated neutrophils can release granule contents extracellularly (degranulation), with potential for collateral tissue damage (2). Platelet-activating factor (PAF), a physiological priming agent capable of substantially enhancing neutrophil degranulation in response to subsequent stimulation (2, 10), has been implicated in COPD pathogenesis and in endothelial damage and remote organ damage in the setting of hypoxia (11, 12). Bacteria release formylated peptides (*N*-formyl-methionyl-leucyl-phenylalanine [fMLP]), which potently activate neutrophils; these peptides are present in cigarette smoke and have been implicated in emphysema progression (13). Patients with COPD suffer recurrent infection-driven exacerbations, but neutrophilic inflammation persists even in the absence of detectable infection, correlating with disease severity and progression (14). Despite evidence of NE-induced lung injury, translation of NE inhibitors has not led to significant benefit (15), perhaps reflecting the complex array of additional neutrophil-secreted proteins with damaging potential.

Patients with COPD have increased risk of cardiovascular morbidity and mortality even after adjusting for shared risk factors, including smoking (16), particularly after exacerbation (17). Pulmonary endothelial dysfunction in patients with COPD can induce pulmonary hypertension, which correlates with hypoxia (18). Accumulating evidence indicates inflammation, oxidative stress, and vascular tissue damage as key mechanisms linking COPD and cardiovascular disease (19), with neutrophil degranulation identified as an important pathway (20). Circulating neutrophils primed for enhanced degranulation have been identified in patients experiencing COPD exacerbation (21), with potential to contribute to endothelial injury.

Herein we show that hypoxia promotes neutrophil degranulation and neutrophil-induced endothelial damage. Proteomic analysis reveals hypoxia-driven secretion of highly histotoxic proteins from healthy neutrophils, and a subset of these are further increased from COPD neutrophils. Supernatants from hypoxic COPD neutrophils enhance neutrophil–endothelial adhesion. Finally, we identify increased concentrations of corresponding hypoxic neutrophil histotoxic granule proteins in COPD plasma, together with endothelial injury biomarkers. Some results have been reported previously in abstract form (22–24).

## Methods

### Ethics Statement

Written informed consent was obtained from all healthy volunteers and patients with COPD (06/Q0108/281, 08/H0308/281, 18/WM/0097, and 20/SS/0085). All studies complied with the Declaration of Helsinki. All animal experiments were approved in accordance with the Animals (Scientific Procedures) Act 1986.

### Human Neutrophils

Venous blood neutrophils were isolated by centrifugation over plasma-Percoll gradients (25). Neutrophils were resuspended in normoxic (atmospheric 21% O_2_) or hypoxic (0.8% O_2_ equating to media Po_2_ of 3 kPa [26], 5% CO_2_, Baker Ruskinn or Whitley hypoxia workstation) Iscove’s modified Dulbecco’s medium (IMDM). At 4 hours, neutrophils (11.1 × 10^6^/ml) were treated with PAF (1 μM, 5 min) and then fMLP (100 nM, 10 min) (2). PI3K inhibitors were added before incubation: PI3Kγ-selective (AS605240, 3 μM) and PI3Kδ-selective (CAL-101, 100 nM).

### Murine Neutrophils

Femoral bone marrow neutrophils were isolated by negative immunomagnetic selection from PI3Kδ-hyperactived E1020K heterozygote (PPL 80/2248 and P4802B8AC) (27), PI3Kδ-kinase-dead D910A homozygote (PPL 70/7661) (28), or PI3Kγ^−/−^ (PPL 70/8100) mice, alongside age- and strain-matched wild-type control mice (E1020K/D910A: C57BL/6J, and PI3Kγ^−/−^: C57BL/E129). Neutrophils were resuspended in normoxic or hypoxic IMDM before treatment at 4 hours with cytochalasin B (5 μg/ml, 5 min) and then fMLP (10 μM, 10 min).

### Protein Secretion

Neutrophil supernatant and plasma protein content were measured by ELISA, chemiluminescence immunoassay, or activity assay. Plasma NE- and PR3-specific fibrinogen cleavage products were measured (1, 29).

### Neutrophil Secretome Preparation for Tandem Mass Tag–Mass Spectrometry

Neutrophils were resuspended in normoxic or hypoxic IMDM (4 h) containing ethylenediaminetetraacetic acid (1 mM) and sivelestat (10 μM) and treated with PAF and fMLP as above. Concentrated protein supernatants underwent tandem mass tag–mass spectrometry (TMT-MS).

### Endothelial Cell Survival

Confluent human pulmonary artery endothelial cells (hPAECs) (Lonza) or human pulmonary microvascular endothelial cells (hPMECs) (Promocell) were treated with neutrophil supernatants, with or without alpha-1-antitrypsin (α1AT, 46 μg/ml, 10 min). Cell detachment of rhodamine phalloidin- and DAPI-stained fixed hPAECs was assessed by immunofluorescence. Viability and apoptosis of unfixed hPAECs or hPMECs was assessed by MTT assay or annexin V positivity.

### Endothelial–Neutrophil Interaction

Confluent hPMECs were treated with neutrophil supernatants. At 4 hours, hPMECs were perfused with neutrophils (1 × 10^6^ cells/ml, 4 min, 0.1 Pa shear stress) and neutrophil adhesion or transmigration assessed.

### Statistical Analysis

Data were analyzed using GraphPad Prism version 9 software, reported as mean ± SEM from (*n*) independent experiments. Gaussian data were analyzed by *t* test or two-way ANOVA with Sidak’s correction. Non-Gaussian data were analyzed by Mann-Whitney test. TMT-MS–generated *P* values were adjusted by Benjamini-Hochberg procedure. A *P* value of less than 0.05 was considered statistically significant.

For further information, *see* Expanded Materials and Methods in the online supplement.

## Results

### Patients Experiencing COPD Exacerbation Have Increased Circulating Evidence of Neutrophil Protease Activity

**Figure 1.**
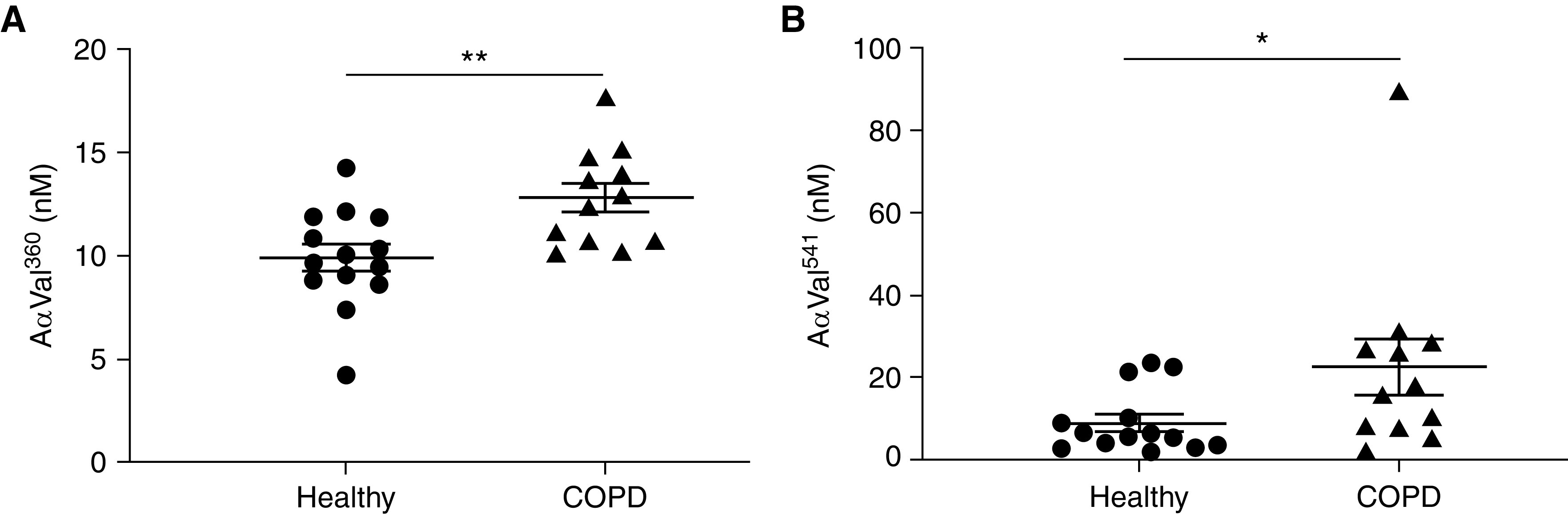
Patients experiencing chronic obstructive pulmonary disease (COPD) exacerbation have a circulating signature indicating increased protease activity. Plasma from patients experiencing COPD exacerbation or age- and sex-matched healthy control subjects was assessed for content of (*A*) neutrophil elastase-specific fibrinogen cleavage product AαVal^360^ or (*B*) PR3-specific fibrinogen cleavage product AαVal^541^ by immunoassay (*n* = 12 COPD, *n* = 14 healthy; cohort 1). Results represent mean ± SEM, (*A*) unpaired *t* test and (*B*) Mann-Whitney test. *P < 0.05 and **P < 0.01.

Although neutrophil proteases are implicated in COPD lung parenchymal destruction, the extent of systemic release of neutrophil granule proteins and their potential role in endothelial injury are unclear. We show that patients experiencing COPD exacerbation (*see* Table E1 in the online supplement) have higher plasma concentrations of fibrinogen cleavage products AαVal^360^ and AαVal^541^ than age- and sex-matched healthy control subjects (Figures 1A and 1B). These footprints specifically indicate increased activity of neutrophil azurophil granule proteases NE and proteinase 3, respectively, secreted on neutrophil activation (which may occur in the circulation, during adherence to vascular endothelium, or after migration into tissues) before inactivation by circulating antiproteases, such as α1AT. Given this systemic signature of enhanced neutrophil protease activity during COPD exacerbation, established evidence of pathological hypoxia during inflammation, and our previous results demonstrating hypoxia-augmented NE release from GM-CSF (granulocyte–macrophage colony–stimulating factor)–primed neutrophils (2), we next examined the ability of inflammatory mediators relevant to COPD and hypoxia to influence neutrophil degranulation.

### Hypoxia Augments NE Release from PAF-primed Neutrophils in a PI3Kγ-Dependent Manner

**Figure 2.**
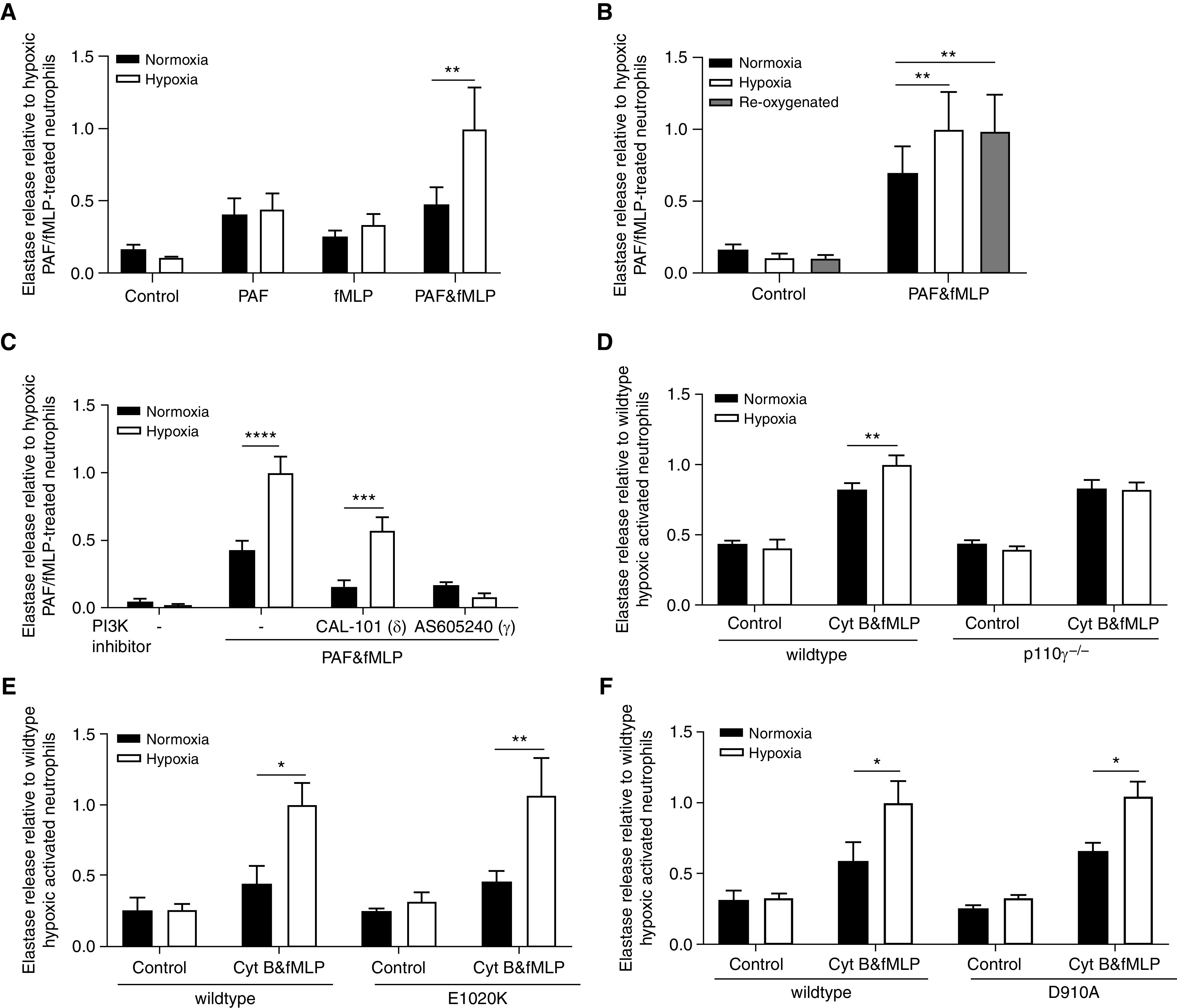
Hypoxia increases elastase release from platelet-activating factor (PAF)-primed neutrophils in a PI3Kγ-dependent manner. (*A*–*C*) Neutrophils from healthy human donors were incubated under normoxia or hypoxia in the presence or absence of PI3Kγ-selective inhibitor (AS605240, 3 μM) or PI3Kδ-selective inhibitor (CAL-101, 100 nM) as indicated. After 4 hours, cells were treated with PAF (1 μM, 5 min) and/or *N*-formyl-methionyl-leucyl-phenylalanine (fMLP) (100 nM, 10 min) or vehicle control as indicated. For reoxygenation, unstimulated hypoxic cells were moved to normoxia with the addition of twice-volume normoxic media for 30 minutes before treatment with PAF and fMLP. Supernatant neutrophil elastase (NE) activity was measured and is expressed as fold change relative to hypoxic activated neutrophils (*A*: *n* = 5, *B*: *n* = 4, *C*: *n* = 4–6). (*D*–*F*) Femoral bone marrow neutrophils were isolated from PI3Kγ-null (PI3Kγ^−/−^), PI3Kδ-hyperactive (E1020K), PI3Kδ-kinase dead (D910A), or wild-type mice from the relevant genetic background. After 4 hours, cells were treated with cytochalasin B (Cyt B; 5 μg/ml, 5 min) and fMLP (10 μM, 10 min) or vehicle control. Supernatant NE activity was measured and is expressed as fold change relative to wild-type hypoxic activated neutrophils (*D*: 3–4 mice per genotype per experiment, *n* = 5 independent experiments; *E* and *F*: 3–4 mice per genotype per experiment, *n* = 3 independent experiments). Results represent mean ± SEM, two-way ANOVA. *P < 0.05, **P < 0.01, ***P < 0.001, and ****P < 0.0001.

Neutrophils treated with PAF and fMLP in combination (but not alone) released up to threefold more active NE when incubated under hypoxia (0.8% O_2_, 3 kPa) compared with normoxia (21 kPa) (Figure 2A). This enhanced secretion was not reversed by reoxygenation (Figure 2B). Given the known role of the PI3K pathway in the hypoxic upregulation of degranulation from GM-CSF–primed neutrophils, and the aberrant chemotaxis of neutrophils from patients with COPD, which could be corrected by PI3K inhibition (30), we explored whether inhibition of PI3K signaling pathways modulated the hypoxic response of PAF-primed neutrophils using PI3K isoform–selective small molecule inhibitors. PI3Kγ-selective inhibition abrogated the hypoxic uplift of NE release from PAF-primed neutrophils; this effect was not seen with the PI3Kδ-selective inhibitor (Figure 2C). These results were replicated in a cohort of patients with COPD (Table E2 and Figure E1). As PI3Kγ inhibition also markedly inhibited NE release from stimulated normoxic cells, we further explored whether PI3K signaling was simply essential for overall degranulation or had a specific role in hypoxia-mediated degranulation, using transgenic mice with abolished or enhanced activity of PI3Kγ/δ isoforms. As PAF did not elicit a detectable priming response in murine neutrophils (data not shown), these cells were treated with cytochalasin B and fMLP to liberate NE. The hypoxic increase in NE release from murine neutrophils deficient in PI3Kγ was abolished, with preserved ability to degranulate under normoxia (Figure 2D). In contrast, the hypoxic enhancement of NE release from murine neutrophils with either activating or kinase-dead PI3Kδ mutations was unaffected (Figures 2E and 2F). Together, these data indicate that the augmented degranulation observed under hypoxia from human or murine neutrophils requires PI3Kγ but not PI3Kδ activity.

### Hypoxia Increases the Capacity for Neutrophil Supernatants to Damage Endothelial Cells

**Figure 3.**
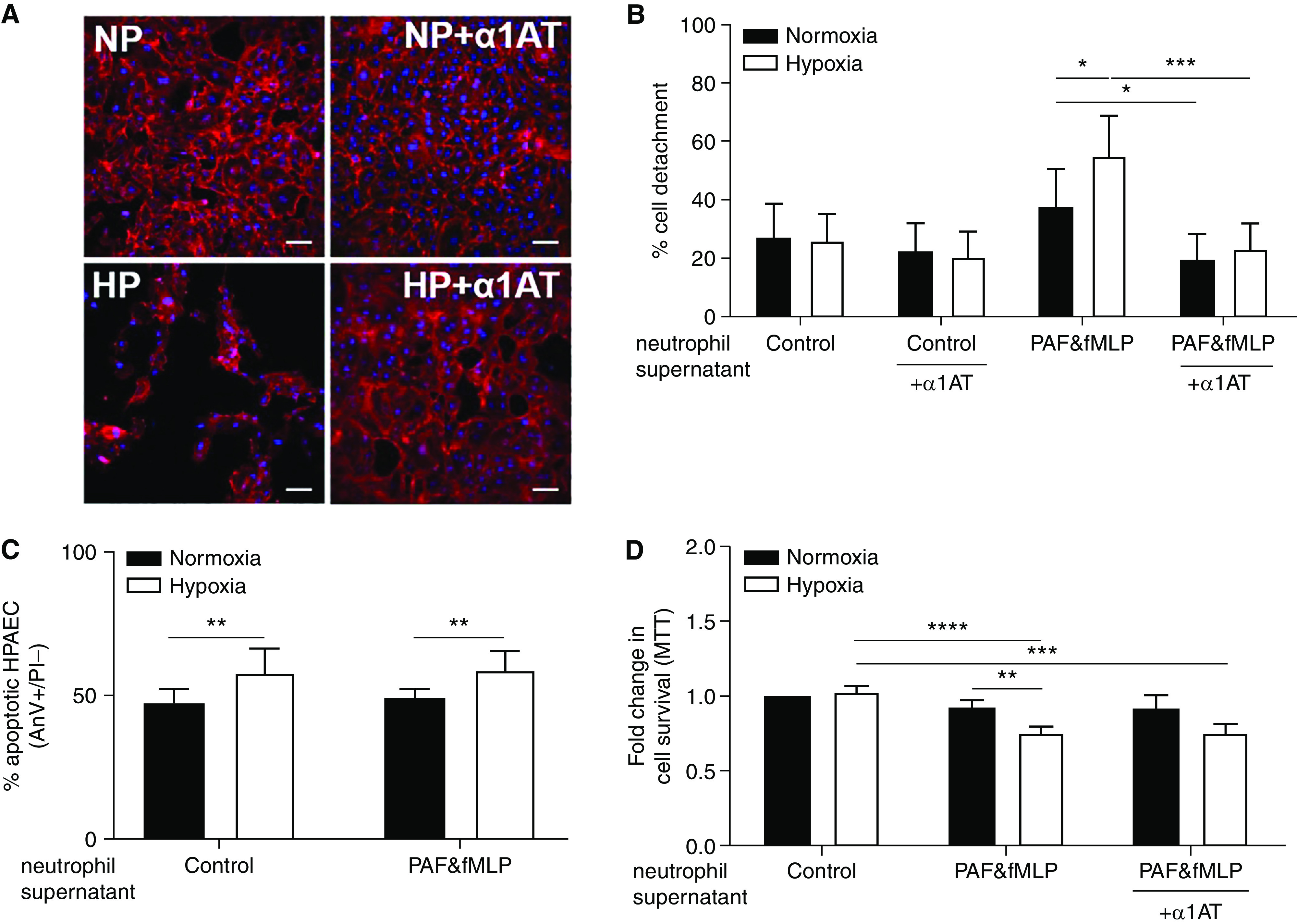
Supernatants from hypoxic activated neutrophils cause increased endothelial cell damage in a partially protease-dependent manner. Neutrophils from healthy donors were incubated under normoxia or hypoxia for 4 hours and then treated with platelet-activating factor (PAF) (1 μM, 5 min) and *N*-formyl-methionyl-leucyl-phenylalanine (fMLP) (100 nM, 10 min) or vehicle control. Supernatants from normoxic versus hypoxic, PAF/fMLP versus vehicle control-treated neutrophils were incubated with confluent human pulmonary artery endothelial cells (hPAECs) for (*A* and *B*) 24 hours, (*C*) 6 hours, or (*D*) 48 hours in the presence or absence of alpha-1-antitrypsin (α1AT, 46 μg/ml) as indicated. (*A* and *B*) hPAECs were fixed and stained with rhodamine-phalloidin and DAPI. Supernatants were from normoxic (NP) or hypoxic (HP) PAF/fMLP-treated neutrophils. Cell detachment was quantified using ImageJ, expressed as percentage detachment of whole field of view. (*A*) Representative confocal images from (*B*) five independent experiments; scale bars, approximately 20 μm. (*C*) hPAECs were stained with FITC-AnV and propidium iodide (PI) for flow cytometric assessment of apoptosis with apoptotic (AnV^+^PI^−^) cells expressed as percentage of total population (*n* = 4). (*D*) Survival of hPAECs was measured by MTT assay (*n* = 6–12). Results represent mean ± SEM, two-way ANOVA. *P < 0.05, **P < 0.01, ***P < 0.001, and ****P < 0.0001. AnV = annexin V; FITC = fluorescein isothiocyanate.

As patients with COPD suffer increased cardiovascular morbidity compared with healthy control subjects and display a footprint of increased circulating protease activity (Figure 1), we investigated whether hypoxia increases the potential for neutrophil-mediated endothelial damage. We incubated supernatants from normoxic versus hypoxic activated (PAF/fMLP) neutrophils with hPAEC and hPMEC monolayers in the presence or absence of α1AT and assessed cell integrity and survival. Supernatants from activated hypoxic neutrophils induced more endothelial detachment (Figures 3A and 3B) and death (Figures 3C, 3D, and E2) than their normoxic counterparts, which was not completely rescued by coincubation with α1AT (Figure 3D).

### Hypoxia Differentially Regulates Protein Release from Activated Neutrophils

**Figure 4.**
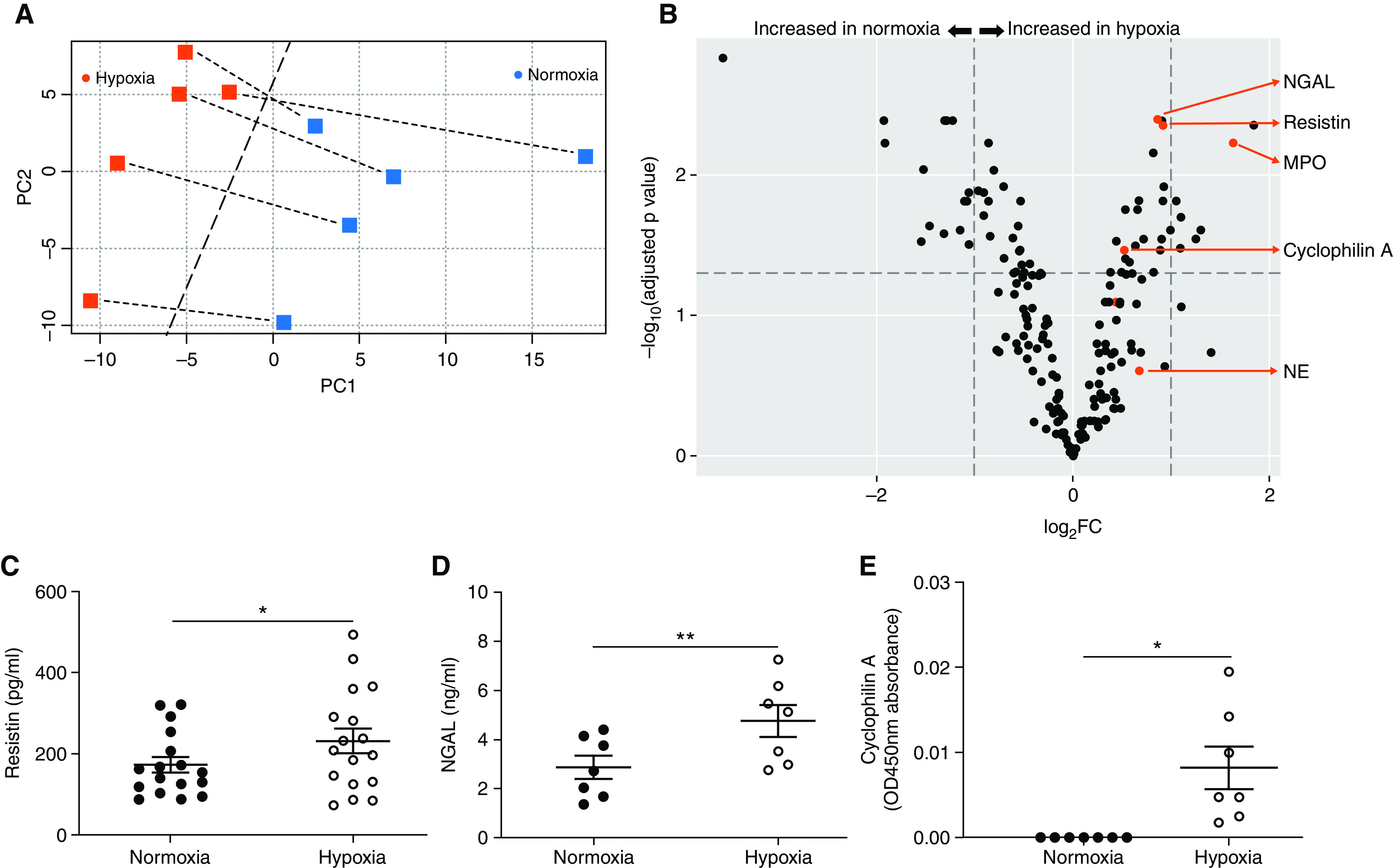
Hypoxia selectively increases granule and cytoplasmic histotoxic protein secretion from activated neutrophils. Neutrophils from healthy donors were incubated under normoxia or hypoxia in the (*A* and *B*) presence or (*C*–*E*) absence of ethylenediaminetetraacetic acid (1 mM) and sivelestat (10 μM) for 4 hours and then treated with platelet-activating factor (PAF) (1 μM, 5 min) and *N*-formyl-methionyl-leucyl-phenylalanine (fMLP) (100 nM, 10 min). (*A* and *B*) Trypsin-digested supernatants were individually labeled with isobaric tags and subjected to tandem mass spectrometry. (*A*) Principal component analysis showed separation of normoxic versus hypoxic supernatant samples by PC1 (dashed line) with samples from individual donors indicated by dashed lines connecting hypoxic and normoxic samples (*n* = 5). (*B*) Volcano plot representation of differential protein expression between paired normoxic and hypoxic supernatants where the vertical dashed lines represent log_2_ fold change (FC) of protein abundance = ±1, and the horizontal dashed line represents adjusted *P* value = 0.05 (*n* = 5). (*C*–*E*) Neutrophil supernatant content of resistin (*C*: *n* = 17), NGAL (neutrophil gelatinase-associated lipocalin) (*D*: *n* = 7), and cyclophilin A (*E*: *n* = 7) was measured from independent samples by ELISA. Results represent mean ± SEM, (*B*) paired *t* test with *P* value adjusted by Benjamini-Hochberg procedure and (*C–E*) paired *t* test. *P < 0.05 and **P < 0.01. MPO = myeloperoxidase; NE = neutrophil elastase; PC = principal component.

Because the antiprotease strategy did not completely mitigate neutrophil-induced endothelial damage (Figure 3D), and multiple neutrophil-derived granule products have potentially damaging actions, we investigated the effect of hypoxia on the total detectable proteome released by activated neutrophils. Mass spectrometry characterization of the normoxic versus hypoxic neutrophil secretome revealed clear separation by principal component analysis (Figure 4A). TMT-MS identified 1,245 proteins, 717 of which were present in all samples. Of these 717 proteins, 199 had a false discovery rate < 0.01, and 63 were differentially regulated (adjusted *P* value < 0.05) between normoxia and hypoxia (Figure 4B). Of these 63 proteins, 35 were more abundant in normoxic (Table 1) and 28 were increased in hypoxic (Table 2) neutrophil supernatants. The majority of proteins upregulated in hypoxic supernatants were granule proteins, whereas those more abundant in normoxic supernatants were predominantly cytoplasmic. However, some granule-associated proteins were increased in normoxic samples (e.g., leukocyte-specific protein 1), and certain cytoplasmic (e.g., cyclophilin A) and nuclear (e.g., histone H4) proteins were increased by hypoxia. Selected hypoxia-upregulated protein targets with the potential to play a role in endothelial damage were biochemically validated using supernatants from independent healthy donors. Concentrations of the azurophil granule protein resistin (Figure 4C), the specific granule protein NGAL (neutrophil gelatinase-associated lipocalin); (Figure 4D), and the cytoplasmic protein cyclophilin A (Figure 4E) were significantly elevated in hypoxic versus normoxic neutrophil supernatants.

**Table 1.** Proteins Significantly Increased in Normoxic Neutrophil Supernatants

Accession	Description	Adjusted *P* Value	FC	Location
Q5TCU8	Tropomyosin β chain	0.001	11.855	CYT
P10599	Thioredoxin	0.017	3.909	CYT
E7EX29	14-3-3 Protein zeta/delta	0.015	3.844	S/G
P52566	Rho GDP-dissociation inhibitor 2	0.004	3.802	CYT
P08670	Vimentin	0.006	3.766	CYT
O00299	Chloride intracellular channel protein 1	0.030	2.918	?
P11021	78-kDa glucose-regulated protein	0.009	2.873	CYT
E9PK25	Cofilin-1	0.023	2.753	CYT
E7EMB3	Calmodulin	0.026	2.488	?
P62993	Growth factor receptor-bound protein 2	0.004	2.478	CYT
P20700	Lamin-B1	0.004	2.435	NUC
Q32MZ4	Leucine-rich repeat flightless-interacting protein 1	0.004	2.337	NUC/CYT
P06737	Glycogen phosphorylase, liver form	0.025	2.217	?
P32942	Intercellular adhesion molecule 3	0.015	2.149	S/G
Q9Y490	Talin-1	0.015	2.116	S/G
O15144	Actin-related protein 2/3 complex subunit 2	0.031	2.086	CYT
P06702	Protein S100-A9	0.013	2.084	CYT
P52209	6-Phosphogluconate dehydrogenase, decarboxylating	0.013	1.950	CYT
P26038	Moesin	0.019	1.878	S
P33241	Leukocyte-specific protein 1	0.013	1.878	S/G
P18206	Vinculin	0.015	1.816	CYT
P30740	Leukocyte elastase inhibitor	0.006	1.813	A
Q96C19	EF-hand domain-containing protein D2	0.027	1.794	?
A6NIZ1	Ras-related protein Rap-1b-like protein	0.009	1.749	SV
P35579	Myosin-9	0.012	1.631	CYT
Q29963	HLA class I histocompatibility antigen, Cw-6 α chain	0.039	1.627	PM
P61247	40S ribosomal protein S3a	0.028	1.528	?
P02042	Hemoglobin subunit delta	0.049	1.492	?
E7EQR4	Ezrin	0.023	1.473	?
P08133	Annexin A6	0.035	1.458	?
P31146	Coronin-1A	0.034	1.451	S/G
Q15907	Ras-related protein Rab-11B	0.015	1.447	G
P46781	40S ribosomal protein S9	0.044	1.438	CYT
P46940	Ras GTPase-activating-like protein IQGAP1	0.049	1.411	G
P39687	Acidic leucine-rich nuclear phosphoprotein 32 family member A	0.043	1.358	?

*Definition of abbreviations*: A = azurophil granules; CYT = cytoplasm; FC = fold change; G = gelatinase granules; NUC = nucleus; PM = plasma membrane; S = specific granules; SV = secretory vesicles.

Proteins significantly increased in supernatants from normoxic neutrophils (adjusted *P* value < 0.05), which were present in all 10 samples with a false-discovery rate < 0.01, are listed in order of the magnitude of the FC. Location data were compiled from Reference 50 and the Uniprot database (www.uniprot.org). For some proteins, the location within neutrophils is currently uncertain or unknown (?).

**Table 2.** Proteins Significantly Increased in Hypoxic Neutrophil Supernatants

Accession	Description	Adjusted *P* Value	FC	Location
P62805	Histone H4	0.004	3.587	NUC
P05164	Myeloperoxidase	0.006	3.107	A
A6NC48	ADP-ribosyl cyclase/cyclic ADP-ribose hydrolase 2	0.025	2.468	SV
O75083	WD repeat-containing protein 1	0.029	2.382	CYT
Q92820	γ-Glutamyl hydrolase	0.020	2.143	S
P02788	Lactotransferrin	0.033	2.136	S
A5A3E0	POTE ankyrin domain family member F	0.015	2.074	CYT
Q0VD83	Apolipoprotein B receptor	0.025	1.993	S/G
P10124	Serglycin	0.012	1.904	CYT
Q9HD89	Resistin	0.004	1.898	A/S
P07737	Profilin-1	0.015	1.890	CYT
A0A087WXL1	Folate receptor γ	0.029	1.877	S
P11215	Integrin α-M	0.004	1.872	S
P16035	Metalloproteinase inhibitor 2	0.034	1.853	G
X6R8F3	Neutrophil gelatinase-associated lipocalin	0.004	1.842	S
V9GYM3	Apolipoprotein A-II	0.049	1.769	?
G3V3D1	Epididymal secretory protein E1	0.007	1.765	A
P10153	Nonsecretory RNase	0.029	1.650	?
P04217	α-1B-glycoprotein	0.015	1.594	?
P05107	Integrin β-2	0.018	1.580	G
P01024	Complement C3	0.032	1.556	?
P20061	Transcobalamin-1	0.042	1.492	S
P78324	Tyrosine-protein phosphatase nonreceptor type substrate 1	0.018	1.449	SV
J3KNB4	Cathelicidin antimicrobial peptide	0.039	1.446	S/G
P62937	Peptidyl-prolyl cis-trans isomerase A/Cyclophilin A	0.035	1.438	CYT
P30086	Phosphatidylethanolamine-binding protein 1	0.049	1.406	?
A0A075B6H6	Ig kappa chain C region	0.030	1.358	?
A0A075B6K9	Ig lambda-2 chain C regions	0.049	1.305	?

*Definition of abbreviations*: A = azurophil granules; CYT = cytoplasm; FC = fold change; G = gelatinase granules; NUC = nucleus; PM = plasma membrane; S = specific granules; SV = secretory vesicles.

Proteins significantly increased in supernatants from hypoxic neutrophils (adjusted *P* value < 0.05), which were present in all 10 samples with a false discovery rate < 0.01, are listed in order of the magnitude of the FC. Location data were compiled from Reference 50 and the Uniprot database (www.uniprot.org). For some proteins, the location within neutrophils is currently uncertain or unknown (?).

### Investigation of Cytoplasmic and Nuclear Protein Secretion

As cyclophilin A is cytoplasmic rather than granule associated, we examined the release of neutrophil-derived microvesicles (NMVs), which contain components derived from parent cells, as a potential source of protein release in addition to degranulation. However, we found no difference in NMV numbers in hypoxic versus normoxic supernatants (Figure E3A) or in plasma from healthy patients versus patients with COPD (Figure E3B). Furthermore, there was no difference in the content of cyclophilin A between NMVs generated from normoxic versus hypoxic cells, and cyclophilin A was also detected in microvesicle-depleted neutrophil supernatants (Figures E3C and E3D).

Because the nuclear protein, histone H4, was increased in the hypoxic supernatant proteome (although other histones were not likewise increased), we also investigated the release of neutrophil extracellular traps (NETs), which release both nuclear and granule proteins into the extracellular space. However, there was no difference in NETosis from normoxic versus hypoxic neutrophils (Figure E4). Overall, our data do not support a contribution of NMVs or NETs to the differential spectrum of cytoplasmic or nuclear proteins released from neutrophils under hypoxia.

### Hypoxic Neutrophils from Patients Experiencing COPD Exacerbation Release More Histotoxic Proteins

**Figure 5.**
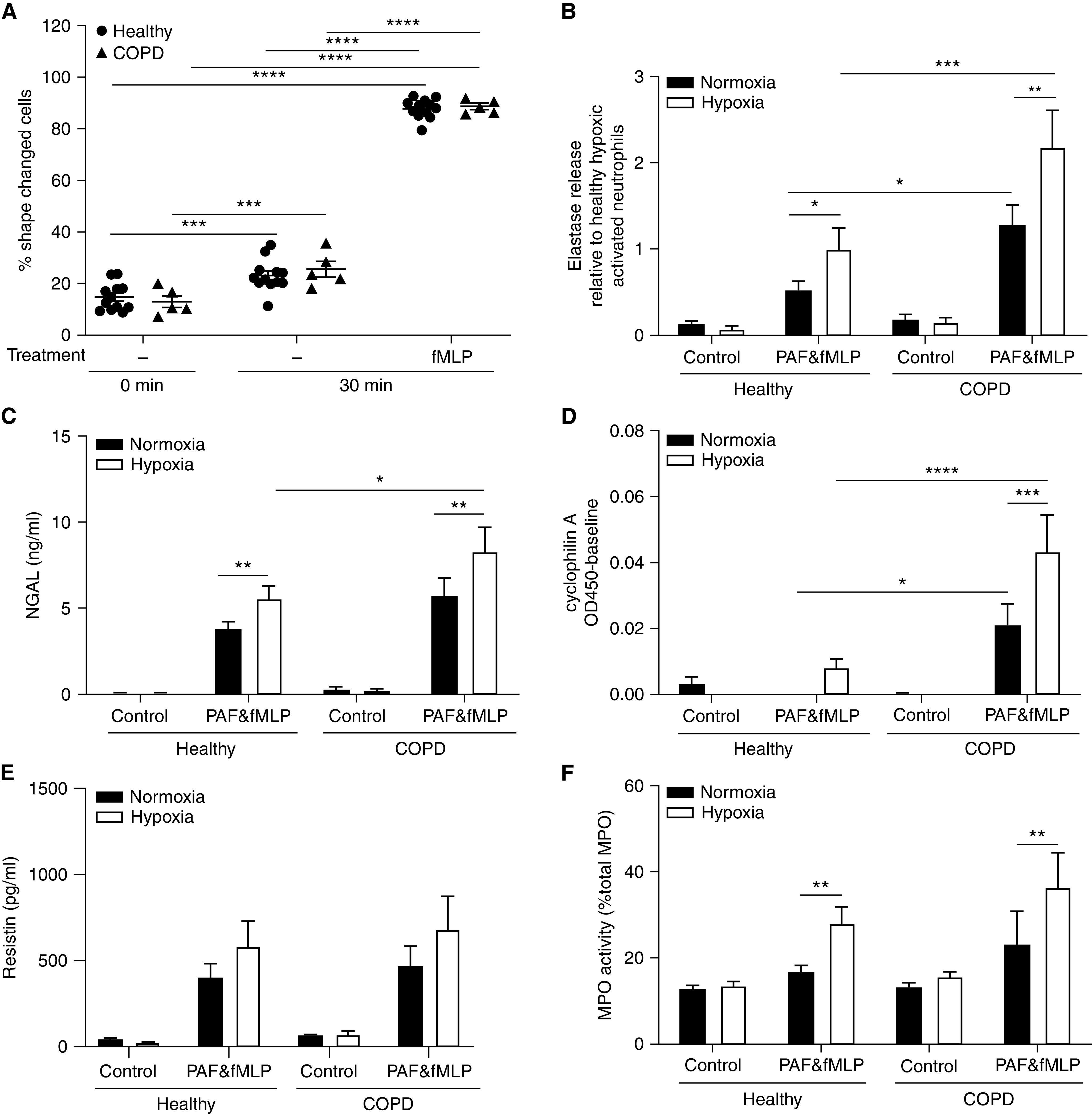
Hypoxia further augments histotoxic protein release from chronic obstructive pulmonary disease (COPD) versus healthy neutrophils. (*A*) Neutrophils from healthy donors or patients experiencing COPD exacerbation were incubated under normoxia and treated with *N*-formyl-methionyl-leucyl-phenylalanine (fMLP) (100 nM, 30 min) or vehicle control. Shape change was assessed by flow cytometric analysis of forward scatter, expressed as percentage shape-changed cells of total population (*n* = 5–12). (*B*–*F*) Neutrophils from healthy donors or patients experiencing COPD exacerbation were incubated under normoxia or hypoxia for 4 hours and then treated with platelet-activating factor (PAF) (1 μM, 5 min) and fMLP (100 nM, 10 min) or vehicle control. Supernatant content of elastase (*B*: *n* = 7–14), NGAL (neutrophil gelatinase-associated lipocalin) (*C*: *n* = 7–12), cyclophilin A (*D*: *n* = 3–7), resistin (*E*: *n* = 7–12), and MPO (myeloperoxidase) (*F*: *n* = 3–6) was measured by ELISA or activity assay. Supernatant neutrophil elastase activity is expressed as fold change relative to healthy hypoxic activated neutrophils. All samples were obtained from cohort 1. Results represent mean ± SEM, two-way ANOVA. *P < 0.05, **P < 0.01, ***P < 0.001, and ****P < 0.0001.

During COPD exacerbations (which are associated with excess cardiovascular morbidity), neutrophils are subject to intensified local and systemic hypoxia in addition to a markedly proinflammatory microenvironment. Because hypoxia enables even “healthy” neutrophils to release multiple proteins capable of causing endothelial damage, we studied the effect of hypoxia on neutrophils isolated from patients experiencing COPD exacerbation (Table E1). COPD neutrophils were not basally shape-changed (indicating no priming or activation) and responded identically to healthy control neutrophils after fMLP stimulation (Figure 5A). Furthermore, there was no difference in the release of NE from unstimulated neutrophils obtained from patients experiencing COPD exacerbation versus age- and sex-matched healthy control subjects under normoxic or hypoxic conditions (Figure 5B). Together, these data indicate that, in our cohort 1, circulating neutrophils from patients with COPD were not primed during exacerbations. Despite this, when incubated under hypoxia, stimulated COPD neutrophils released up to threefold more active NE than equivalent healthy control cells (Figure 5B). Likewise, secretion of selected granule and cytoplasmic protein targets identified by proteomics NGAL (Figure 5C) and cyclophilin A (Figure 5D) was increased 1.5- and 5-fold, respectively, from stimulated hypoxic neutrophils from patients with COPD versus healthy control subjects, with a similar pattern demonstrated for resistin release (Figure 5E). In contrast, although secretion of the azurophil granule protein MPO (myeloperoxidase) was consistently increased in hypoxia versus normoxia, it was not further enhanced when comparing COPD and healthy control neutrophils (Figure 5F).

### Hypoxia Promotes Endothelial–Neutrophil Interaction Induced by COPD Patient Neutrophil Supernatants

**Figure 6.**
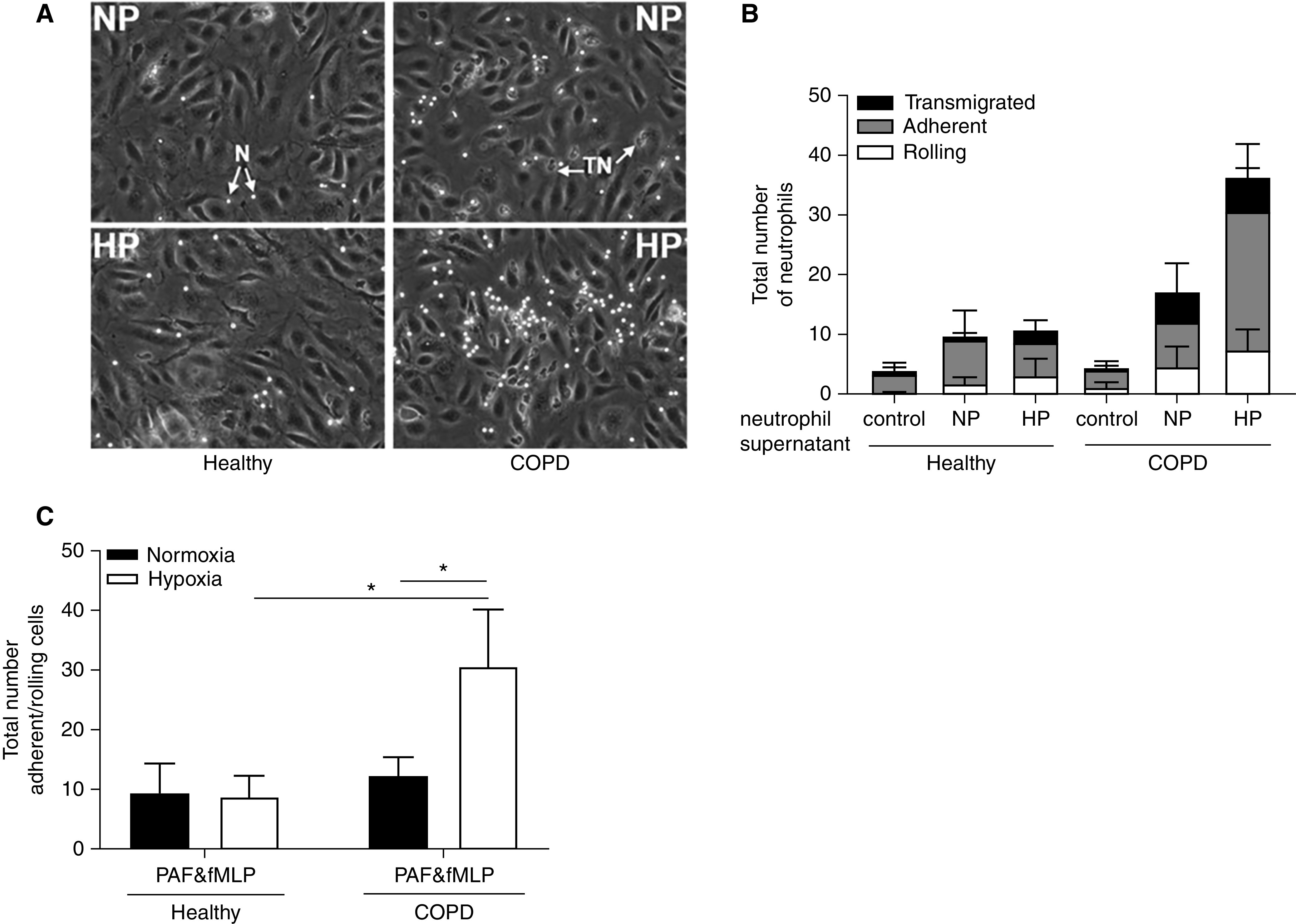
Hypoxia accentuates endothelial–neutrophil interaction induced by chronic obstructive pulmonary disease (COPD) versus healthy neutrophil supernatants. Neutrophils from healthy donors or patients experiencing COPD exacerbation were incubated under normoxia or hypoxia for 4 hours and then treated with platelet-activating factor (PAF) (1 μM, 5 min) and *N*-formyl-methionyl-leucyl-phenylalanine (fMLP) (100 nM, 10 min). Supernatants from normoxic (NP) or hypoxic (HP) PAF- or fMLP-treated neutrophils were incubated with confluent human pulmonary microvascular endothelial cells (hPMECs) for 4 hours in the presence of serum (2%). Washed hPMECs were perfused with healthy neutrophils. (*A*) Representative images (original magnification, ×100) showing arrested/rolling neutrophils (N) as bright phase and transmigrated neutrophils (TN) as dark phase. (*B*) Endothelial–neutrophil interactions (total number of rolling, adhered, and transmigrated neutrophils after bolus neutrophil injection) were captured with time-lapse imaging (*n* = 3). (*C*) Quantification of neutrophil rolling and adherence was performed using ImagePro software (*n* = 3). All neutrophil supernatant samples were obtained from cohort 1. Results represent mean ± SEM, two-way ANOVA. *P < 0.05.

To investigate whether neutrophils from patients with COPD have increased capacity for endothelial cell injury and/or activation, we applied supernatants from normoxic or hypoxic activated COPD versus healthy neutrophils to hPMEC monolayers and assessed neutrophil recruitment (rolling, adhesion, and transmigration) in a biologically relevant *in vitro* flow system (Figures 6A and 6B). Treatment with hypoxic COPD neutrophil supernatants resulted in a marked increase in neutrophil rolling and adhesion compared with both normoxic COPD supernatants and hypoxic healthy supernatants (Figure 6C).

### Hypoxia May Synergize with Inflammatory Mediators to Promote Upregulation of Circulating Histotoxic Neutrophil Granule Proteins in Patients with COPD

**Figure 7.**
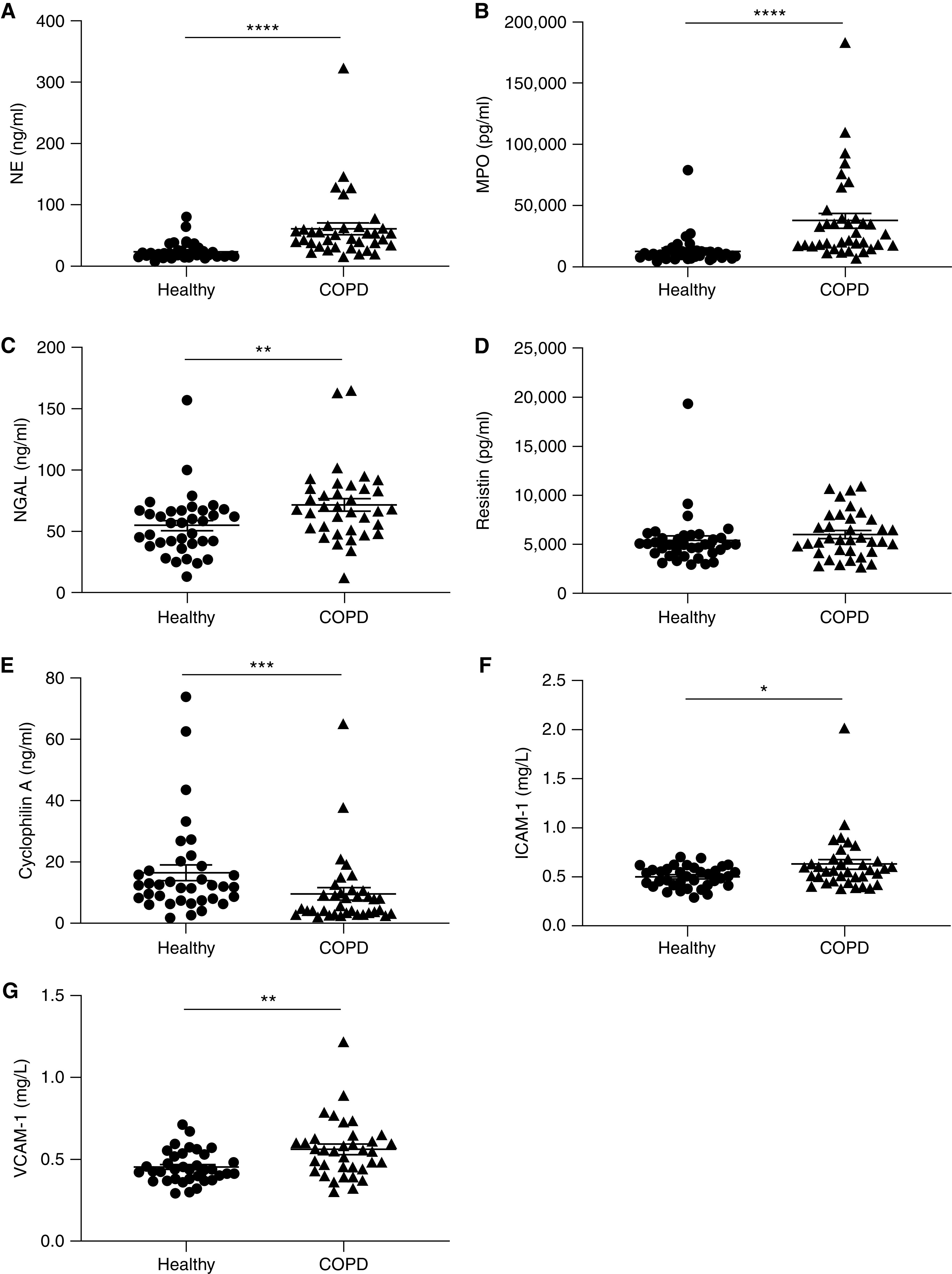
Plasma from patients with chronic obstructive pulmonary disease (COPD) has increased content of hypoxia-upregulated histotoxic granule proteins and vascular injury biomarkers. Plasma from healthy donors or patients experiencing COPD exacerbation was assessed for content of (*A*) neutrophil elastase (NE), (*B*) MPO (myeloperoxidase), (*C*) NGAL (neutrophil gelatinase-associated lipocalin), (*D*) resistin, (*E*) cyclophilin A, (*F*) ICAM-1 (intercellular adhesion molecule-1), and (*G*) VCAM-1 (vascular cell adhesion molecule-1) by ELISA (NE, NGAL, and cyclophilin A) or chemiluminescence immunoassay (MPO, resistin, ICAM-1, and VCAM-1) (*n* = 36 healthy, *n* = 36 COPD; 4 samples from cohort 1 and 32 samples from cohort 3). Results represent mean ± SEM, Mann-Whitney test. *P < 0.05, **P < 0.01, ***P < 0.001, and ****P < 0.0001.

Because hypoxia enhanced histotoxic protein release from COPD neutrophils (Figure 5), and supernatants from these cells promoted neutrophil–endothelial interaction (Figure 6), we examined whether there was a circulating signature of hypoxia-induced neutrophil protein secretion. We detected significantly increased concentrations of the neutrophil granule proteins NE (Figure 7A), MPO (Figure 7B), and NGAL (Figure 7C) in patients experiencing COPD exacerbation versus healthy control plasma (derived from an independent cohort 3) (Table E3), although there was no difference in the plasma content of resistin (Figure 7D). Despite the observation of increased release of the cytoplasmic protein cyclophilin A from isolated COPD neutrophils (Figure 5E), unexpectedly, the plasma content of cyclophilin A was higher for healthy control subjects than patients with COPD (Figure 7E). Biomarkers of vascular injury or activation ICAM-1 (intercellular adhesion molecule-1) (Figure 7F) and VCAM-1 (vascular cell adhesion molecule-1) (Figure 7G) and inflammation (Figures E5A and E5B) were increased in plasma from patients experiencing COPD exacerbation. In contrast, the COPD plasma content of angiogenesis biomarkers was predominantly unchanged compared with that of healthy control subjects (Figures E5C–E5J), suggesting a damage phenotype that may enhance endothelial–neutrophil interaction but without a corresponding increase in vascular regeneration ability.

## Discussion

Our work demonstrates that hypoxic neutrophils display a hypersecretory phenotype with enhanced capacity to both activate and injure cultured endothelial cells. Hypoxia-driven release of histotoxic proteins was observed from healthy donor neutrophils and translated to a pathophysiologically relevant cohort of patients experiencing COPD exacerbation, confirming even further augmented histotoxic protein secretion under hypoxia, together with higher circulating concentrations of selected neutrophil-derived proteins and a plasma signature of increased neutrophil protease activity and vascular injury.

Our data from human neutrophils treated with PI3K isoform-selective inhibitors and from transgenic murine cells support a nonredundant role for PI3Kγ in the hypoxic augmentation of NE release. Dysregulated PI3K signaling has previously been associated with COPD, with the impaired neutrophil migratory accuracy improved by PI3Kγ/δ inhibition (30), although we found no role for the δ isoform in hypoxic degranulation. Our results suggest that PI3Kγ is required for hypoxia-potentiated neutrophil degranulation, whether in response to tyrosine kinase– (2) or G-protein–coupled agonists, such as PAF. Consistent with a role for PI3Kγ-dependent PAF/hypoxia-mediated neutrophil degranulation in vascular insults, PI3Kγ/δ inhibition limited both PAF-induced hindlimb inflammation and ischemic cardiac infarct size (31), and PI3Kγ-null mice had improved cardiac recovery after ischemia (32). Hence, this signaling pathway could conceivably be targeted to mitigate neutrophil-mediated endothelial damage in COPD and other diseases underpinned by hypoxia, inflammation, and vascular damage.

Hypoxia enhances neutrophil degranulation, but, surprisingly, hypoxia-upregulated proteins identified by proteomic analysis did not segregate precisely with discrete granule populations. We have previously shown that hypoxia promotes differential secretion from eosinophil granules (26), with similar results reported using mast cells (33). Our results imply a comparable “differential degranulation” may be occurring from neutrophils in the setting of hypoxia. A limited number of studies analyzing the neutrophil secretome generated under normoxic conditions have shown variation in protein content according to the inciting stimulus (34), suggesting that the (potentially hypoxic) inflammatory environment can influence the precise composition of secreted granule proteins. This may also explain our observation that release of the azurophilic granule proteins NE, resistin, and MPO from hypoxic COPD neutrophils did not fully mirror each other.

In addition to enhanced degranulation, our proteomic data further suggest active secretion of selected cytoplasmic and nuclear proteins under hypoxia. We provide the first description of neutrophilic secretion of the proinflammatory cytoplasmic protein cyclophilin A, aligning to a previous study demonstrating its hypoxia-driven secretion from cardiac myocytes (35). Our data also demonstrated increased release of the nuclear protein histone H4 (although no other histones) in hypoxic supernatants. The source of this protein remains unclear, as we did not observe any difference in NETosis between normoxic and hypoxic neutrophils.

We have established that peripheral blood neutrophils from patients experiencing COPD exacerbation display markedly greater hypoxic release of NE, NGAL, and cyclophilin A relative to matched healthy control subjects. These proteins have been previously implicated in endothelial dysfunction or atherosclerosis (36, 37), with raised circulating levels of NGAL associated with cardiovascular events and with hypoxemia in COPD (38, 39). Although we found no relationship between admission or venesection oxygen levels and protein release (data not shown), this may have been confounded by prior exposure to supplemental oxygen. In keeping with a potential *in vivo* role for hypoxic augmentation of neutrophil degranulation, we found higher levels of the granule proteins NE, MPO, and NGAL and a footprint of increased NE and PR3 activity in COPD versus healthy plasma, aligning with recent severe acute respiratory syndrome coronavirus 2 (SARS-CoV-2) studies, where NGAL in particular was associated with mortality (40). Our results suggest that enhanced neutrophil degranulation in the setting of hypoxia may contribute to systemic endothelial injury in patients experiencing COPD exacerbation and potentially other conditions, such as SARS-CoV-2 infection.

Primed circulating neutrophils with heightened potential for cellular damage have been identified in patients with COPD (21). However, we did not observe significant shape change or enhanced degranulation (both features of priming) from unstimulated COPD neutrophils, suggesting that the enhanced hypoxic release of granule proteins from these cells is not simply a consequence of priming and may “substitute” for this process in promoting damage. A potential explanation for enhanced protein release is a change in protein abundance in COPD neutrophils, with one study showing increased NE activity in COPD versus healthy neutrophil lysates (41). However, this result may represent a protease/antiprotease imbalance, as leukocyte elastase inhibitor was reduced, a finding consistent with our proteomic data and that may explain the discrepancy between these results and the NE activity assay.

Our study has some limitations. Although our patients and control subjects were age and sex matched, the patients had a significant smoking history and more comorbidities than the healthy volunteers. The patients with COPD were studied during exacerbations, a high-risk period for acute cardiovascular events (17), but with variation in terms of exacerbation etiology and severity; however, the use of two separate cohorts in different institutions mitigates this limitation. It is possible that acute or long-term medications could affect neutrophil function in our patient cohorts. For example, all patients with COPD in cohort 1 were taking inhaled combination corticosteroid and long-acting β-agonists and were treated with oral prednisolone during exacerbation with varying duration before venesection. However, although glucocorticoids are known to delay neutrophil apoptosis, this effect is lost in hypoxia (42), and inhaled corticosteroids have been shown not to affect neutrophil protein secretion (43). Given the variation in comorbidities (Table E4), and thus treatments, within our cohorts, there is unlikely to be any consistent impact on neutrophil function.

Although our results are predominantly consistent with previous studies, we note that despite reports of enhanced circulating levels of resistin and cyclophilin A in patients with COPD versus healthy control subjects (44, 45), we did not detect such an increase in our COPD cohort. These discrepant results may reflect differences in the timing of sampling, medications, patient heterogeneity, or obscuration of a neutrophil-specific signal, because both proteins have multiple cellular sources (46, 47). Both cyclophilin A and its receptor have been detected at high levels in atherosclerotic plaques (48). Hence, in COPD, cyclophilin A may already be membrane bound, preventing its detection in plasma. As patients with COPD have increased atherosclerotic burden, hypoxia within plaques may promote enhanced local release of cyclophilin A from neutrophils in this and other microenvironments. This would be consistent with our *in vitro* data and with a previous study demonstrating increased cyclophilin A expression in the lung tissue of patients with COPD compared with either smoking or nonsmoking control subjects (49).

Overall, our data demonstrate that patients experiencing COPD exacerbation have an enhanced footprint of circulating neutrophil protease activity and that neutrophils from these patients exhibit a hypoxia-driven hypersecretory phenotype with enhanced capacity for endothelial damage. We provide the first description of the ability of hypoxia to augment the secretion of histotoxic proteins from COPD neutrophils *in vitro* and have identified a corresponding increase in the plasma concentrations of selected granule proteins and markers of vascular injury in patients with COPD. Our findings may illuminate novel therapeutic targets in treatment-recalcitrant neutrophil-mediated inflammatory diseases such as COPD.

## References

[1] Carter RI Ungurs MJ Mumford RA Stockley RA Aα-Val360: a marker of neutrophil elastase and COPD disease activity *Eur Respir J* 2013 41 31 38 22523359 10.1183/09031936.00197411

[2] Hoenderdos K Lodge KM Hirst RA Chen C Palazzo SG Emerenciana A *et al.* Hypoxia upregulates neutrophil degranulation and potential for tissue injury *Thorax* 2016 71 1030 1038 27581620 10.1136/thoraxjnl-2015-207604 PMC5099189

[3] Nolte D Steinhauser P Pickelmann S Berger S Härtl R Messmer K Effects of diaspirin-cross-linked hemoglobin (DCLHb) on local tissue oxygen tension in striated skin muscle: an efficacy study in the hamster *J Lab Clin Med* 1997 130 328 338 9341993 10.1016/s0022-2143(97)90028-7

[4] van Staveren S Ten Haaf T Klöpping M Hilvering B Tinnevelt GH de Ruiter K *et al.* Multi-dimensional flow cytometry analysis reveals increasing changes in the systemic neutrophil compartment during seven consecutive days of endurance exercise *PLoS One* 2018 13 e0206175 30376575 10.1371/journal.pone.0206175 PMC6207321

[5] Mall MA Harkema JR Trojanek JB Treis D Livraghi A Schubert S *et al.* Development of chronic bronchitis and emphysema in β-epithelial Na+ channel-overexpressing mice *Am J Respir Crit Care Med* 2008 177 730 742 18079494 10.1164/rccm.200708-1233OC PMC2277210

[6] Belton M Brilha S Manavaki R Mauri F Nijran K Hong YT *et al.* Hypoxia and tissue destruction in pulmonary TB *Thorax* 2016 71 1145 1153 27245780 10.1136/thoraxjnl-2015-207402 PMC5136721

[7] Polosukhin VV Cates JM Lawson WE Milstone AP Matafonov AG Massion PP *et al.* Hypoxia-inducible factor-1 signalling promotes goblet cell hyperplasia in airway epithelium *J Pathol* 2011 224 203 211 21557221 10.1002/path.2863 PMC5901746

[8] Joshi FR Manavaki R Fryer TD Figg NL Sluimer JC Aigbirhio FI *et al.* Vascular imaging with 18F-fluorodeoxyglucose positron emission tomography is influenced by hypoxia *J Am Coll Cardiol* 2017 69 1873 1874 28385317 10.1016/j.jacc.2017.01.050 PMC5380109

[9] McElvaney OJ McEvoy NL McElvaney OF Carroll TP Murphy MP Dunlea DM *et al.* Characterization of the inflammatory response to severe COVID-19 illness *Am J Respir Crit Care Med* 2020 202 812 821 32584597 10.1164/rccm.202005-1583OC PMC7491404

[10] Kitchen E Rossi AG Condliffe AM Haslett C Chilvers ER Demonstration of reversible priming of human neutrophils using platelet-activating factor *Blood* 1996 88 4330 4337 8943870

[11] Bitencourt CS Bessi VL Huynh DN Ménard L Lefebvre JS Lévesque T *et al.* Cooperative role of endogenous leucotrienes and platelet-activating factor in ischaemia-reperfusion-mediated tissue injury *J Cell Mol Med* 2013 17 1554 1565 24373549 10.1111/jcmm.12118 PMC3914641

[12] Shukla SD Muller HK Latham R Sohal SS Walters EH Platelet-activating factor receptor (PAFr) is upregulated in small airways and alveoli of smokers and COPD patients *Respirology* 2016 21 504 510 26662379 10.1111/resp.12709

[13] Cardini S Dalli J Fineschi S Perretti M Lungarella G Lucattelli M Genetic ablation of the fpr1 gene confers protection from smoking-induced lung emphysema in mice *Am J Respir Cell Mol Biol* 2012 47 332 339 22461430 10.1165/rcmb.2012-0036OC

[14] Stănescu D Sanna A Veriter C Kostianev S Calcagni PG Fabbri LM *et al.* Airways obstruction, chronic expectoration, and rapid decline of FEV_1_ in smokers are associated with increased levels of sputum neutrophils *Thorax* 1996 51 267 271 8779129 10.1136/thx.51.3.267 PMC1090637

[15] Kuna P Jenkins M O’Brien CD Fahy WA AZD9668, a neutrophil elastase inhibitor, plus ongoing budesonide/formoterol in patients with COPD *Respir Med* 2012 106 531 539 22197578 10.1016/j.rmed.2011.10.020

[16] Ye C Younus A Malik R Roberson L Shaharyar S Veledar E *et al.* Subclinical cardiovascular disease in patients with chronic obstructive pulmonary disease: a systematic review *QJM* 2017 110 341 349 27539486 10.1093/qjmed/hcw135

[17] Kunisaki KM Dransfield MT Anderson JA Brook RD Calverley PMA Celli BR *et al.* SUMMIT Investigators Exacerbations of chronic obstructive pulmonary disease and cardiac events: a *post hoc* cohort analysis from the SUMMIT randomized clinical trial *Am J Respir Crit Care Med* 2018 198 51 57 29442524 10.1164/rccm.201711-2239OC PMC6913068

[18] Dinh-Xuan AT Higenbottam TW Clelland CA Pepke-Zaba J Cremona G Butt AY *et al.* Impairment of endothelium-dependent pulmonary-artery relaxation in chronic obstructive lung disease *N Engl J Med* 1991 324 1539 1547 2027358 10.1056/NEJM199105303242203

[19] Eickhoff P Valipour A Kiss D Schreder M Cekici L Geyer K *et al.* Determinants of systemic vascular function in patients with stable chronic obstructive pulmonary disease *Am J Respir Crit Care Med* 2008 178 1211 1218 18836149 10.1164/rccm.200709-1412OC

[20] Shi Y Yang S Luo M Zhang W-D Ke Z-P Systematic analysis of coronary artery disease datasets revealed the potential biomarker and treatment target *Oncotarget* 2017 8 54583 54591 28903366 10.18632/oncotarget.17426 PMC5589605

[21] Oudijk E-JD Gerritsen WBM Nijhuis EHJ Kanters D Maesen BLP Lammers J-WJ *et al.* Expression of priming-associated cellular markers on neutrophils during an exacerbation of COPD *Respir Med* 2006 100 1791 1799 16531033 10.1016/j.rmed.2006.01.022

[22] Lodge K Hoenderdos K Robbins A Storisteanu D Chilvers E Li W *et al.* Hypoxia upregulates PI3Kinase-dependent neutrophil degranulation and neutrophil-mediated tissue injury [abstract] *Thorax* 2016 71 A27 10.1136/thoraxjnl-2015-207604 PMC5099189 27581620

[23] Lodge K Hoenderdos K Chilvers E Li W Condliffe A Hypoxia upregulates PI3Kinase-dependent neutrophil degranulation and neutrophil-mediated tissue injury [abstract] *Am J Respir Crit Care Med* 2017 195 A7058

[24] Lodge K Hoenderdos K Robbins A Chilvers E Li W Condliffe A Hypoxia drives neutrophil-mediated endothelial damage in COPD [abstract] *Thorax* 2017 72 A69 A70

[25] Haslett C Guthrie LA Kopaniak MM Johnston RB Jr Henson PM Modulation of multiple neutrophil functions by preparative methods or trace concentrations of bacterial lipopolysaccharide *Am J Pathol* 1985 119 101 110 2984939 PMC1888083

[26] Porter LM Cowburn AS Farahi N Deighton J Farrow SN Fiddler CA *et al.* Hypoxia causes IL-8 secretion, Charcot Leyden crystal formation, and suppression of corticosteroid-induced apoptosis in human eosinophils *Clin Exp Allergy* 2017 47 770 784 28000962 10.1111/cea.12877

[27] Stark A-K Chandra A Chakraborty K Alam R Carbonaro V Clark J *et al.* PI3Kδ hyper-activation promotes development of B cells that exacerbate *Streptococcus pneumoniae* infection in an antibody-independent manner *Nat Commun* 2018 9 3174 30093657 10.1038/s41467-018-05674-8 PMC6085315

[28] Okkenhaug K Bilancio A Farjot G Priddle H Sancho S Peskett E *et al.* Impaired B and T cell antigen receptor signaling in p110δ PI 3-kinase mutant mice *Science* 2002 297 1031 1034 12130661 10.1126/science.1073560

[29] Newby PR Crossley D Crisford H Stockley JA Mumford RA Carter RI *et al.* A specific proteinase 3 activity footprint in α_1_-antitrypsin deficiency *ERJ Open Res* 2019 5 00095 02019 31403052 10.1183/23120541.00095-2019 PMC6680069

[30] Sapey E Stockley JA Greenwood H Ahmad A Bayley D Lord JM *et al.* Behavioral and structural differences in migrating peripheral neutrophils from patients with chronic obstructive pulmonary disease *Am J Respir Crit Care Med* 2011 183 1176 1186 21257786 10.1164/rccm.201008-1285OC

[31] Doukas J Wrasidlo W Noronha G Dneprovskaia E Fine R Weis S *et al.* Phosphoinositide 3-kinase γ/δ inhibition limits infarct size after myocardial ischemia/reperfusion injury *Proc Natl Acad Sci USA* 2006 103 19866 19871 17172449 10.1073/pnas.0606956103 PMC1702529

[32] Alloatti G Levi R Malan D Del Sorbo L Bosco O Barberis L *et al.* Phosphoinositide 3-kinase γ-deficient hearts are protected from the PAF-dependent depression of cardiac contractility *Cardiovasc Res* 2003 60 242 249 14613853 10.1016/j.cardiores.2003.08.008

[33] Möllerherm H Branitzki-Heinemann K Brogden G Elamin AA Oehlmann W Fuhrmann H *et al.* Hypoxia modulates the response of mast cells to *Staphylococcus aureus* infection *Front Immunol* 2017 8 541 28553287 10.3389/fimmu.2017.00541 PMC5425595

[34] Snäll J Linnér A Uhlmann J Siemens N Ibold H Janos M *et al.* Differential neutrophil responses to bacterial stimuli: Streptococcal strains are potent inducers of heparin-binding protein and resistin-release *Sci Rep* 2016 6 21288 26887258 10.1038/srep21288 PMC4758080

[35] Seko Y Fujimura T Taka H Mineki R Murayama K Nagai R Hypoxia followed by reoxygenation induces secretion of cyclophilin A from cultured rat cardiac myocytes *Biochem Biophys Res Commun* 2004 317 162 168 15047162 10.1016/j.bbrc.2004.03.021

[36] Tian-Tian Z Jun-Feng Z Heng G Functions of cyclophilin A in atherosclerosis *Exp Clin Cardiol* 2013 18 e118 e124 23940449 PMC3718612

[37] Wen G An W Chen J Maguire EM Chen Q Yang F *et al.* Genetic and pharmacologic inhibition of the neutrophil elastase inhibits experimental atherosclerosis *J Am Heart Assoc* 2018 7 e008187 29437605 10.1161/JAHA.117.008187 PMC5850208

[38] Lindberg S Pedersen SH Mogelvang R Jensen JS Flyvbjerg A Galatius S *et al.* Prognostic utility of neutrophil gelatinase-associated lipocalin in predicting mortality and cardiovascular events in patients with ST-segment elevation myocardial infarction treated with primary percutaneous coronary intervention *J Am Coll Cardiol* 2012 60 339 345 22813613 10.1016/j.jacc.2012.04.017

[39] Eagan TM Damås JK Ueland T Voll-Aanerud M Mollnes TE Hardie JA *et al.* Neutrophil gelatinase-associated lipocalin: a biomarker in COPD *Chest* 2010 138 888 895 20495108 10.1378/chest.09-2718

[40] Abers MS Delmonte OM Ricotta EE Fintzi J Fink DL de Jesus AAA *et al.* NIAID COVID-19 Consortium An immune-based biomarker signature is associated with mortality in COVID-19 patients *JCI Insight* 2021 6 e144455 33232303 10.1172/jci.insight.144455 PMC7821609

[41] Shahriary A Mehrani H Ghanei M Parvin S Comparative proteome analysis of peripheral neutrophils from sulfur mustard-exposed and COPD patients *J Immunotoxicol* 2015 12 132 139 24852194 10.3109/1547691X.2014.914110

[42] Marwick JA Dorward DA Lucas CD Jones KO Sheldrake TA Fox S *et al.* Oxygen levels determine the ability of glucocorticoids to influence neutrophil survival in inflammatory environments *J Leukoc Biol* 2013 94 1285 1292 23964116 10.1189/jlb.0912462 PMC3855024

[43] Culpitt SV Maziak W Loukidis S Nightingale JA Matthews JL Barnes PJ Effect of high dose inhaled steroid on cells, cytokines, and proteases in induced sputum in chronic obstructive pulmonary disease *Am J Respir Crit Care Med* 1999 160 1635 1639 10556133 10.1164/ajrccm.160.5.9811058

[44] Kumor-Kisielewska A Kierszniewska-Stępień D Pietras T Kroczyńska-Bednarek J Kurmanowska Z Antczak A *et al.* Assessment of leptin and resistin levels in patients with chronic obstructive pulmonary disease *Pol Arch Med Wewn* 2013 123 215 220 23611920

[45] Zhang M Tang J Yin J Wang X Feng X Yang X *et al.* The clinical implication of serum cyclophilin A in patients with chronic obstructive pulmonary disease *Int J Chron Obstruct Pulmon Dis* 2018 13 357 363 29403273 10.2147/COPD.S152898 PMC5783015

[46] Park HK Ahima RS Resistin in rodents and humans *Diabetes Metab J* 2013 37 404 414 24404511 10.4093/dmj.2013.37.6.404 PMC3881324

[47] Bukrinsky M Extracellular cyclophilins in health and disease *Biochim Biophys Acta* 2015 1850 2087 2095 25445705 10.1016/j.bbagen.2014.11.013 PMC4436085

[48] Wang C Jin R Zhu X Yan J Li G Function of CD147 in atherosclerosis and atherothrombosis *J Cardiovasc Transl Res* 2015 8 59 66 25604960 10.1007/s12265-015-9608-6 PMC4351140

[49] Hu R Ouyang Q Dai A Tan S Xiao Z Tang C Heat shock protein 27 and cyclophilin A associate with the pathogenesis of COPD *Respirology* 2011 16 983 993 21585617 10.1111/j.1440-1843.2011.01993.x

[50] Lominadze G Powell DW Luerman GC Link AJ Ward RA McLeish KR Proteomic analysis of human neutrophil granules *Mol Cell Proteomics* 2005 4 1503 1521 15985654 10.1074/mcp.M500143-MCP200

